# Nucleotide pools dictate the identity and frequency of ribonucleotide incorporation in mitochondrial DNA

**DOI:** 10.1371/journal.pgen.1006628

**Published:** 2017-02-16

**Authors:** Anna-Karin Berglund, Clara Navarrete, Martin K. M. Engqvist, Emily Hoberg, Zsolt Szilagyi, Robert W. Taylor, Claes M. Gustafsson, Maria Falkenberg, Anders R. Clausen

**Affiliations:** 1 Institute of Biomedicine, University of Gothenburg, Gothenburg, Sweden; 2 Wellcome Trust Centre for Mitochondrial Research, Institute of Neuroscience, The Medical School, Newcastle University, Newcastle upon Tyne, United Kingdom; Max Planck Institute for Biology of Ageing, GERMANY

## Abstract

Previous work has demonstrated the presence of ribonucleotides in human mitochondrial DNA (mtDNA) and in the present study we use a genome-wide approach to precisely map the location of these. We find that ribonucleotides are distributed evenly between the heavy- and light-strand of mtDNA. The relative levels of incorporated ribonucleotides reflect that DNA polymerase γ discriminates the four ribonucleotides differentially during DNA synthesis. The observed pattern is also dependent on the mitochondrial deoxyribonucleotide (dNTP) pools and disease-causing mutations that change these pools alter both the absolute and relative levels of incorporated ribonucleotides. Our analyses strongly suggest that DNA polymerase γ-dependent incorporation is the main source of ribonucleotides in mtDNA and argues against the existence of a mitochondrial ribonucleotide excision repair pathway in human cells. Furthermore, we clearly demonstrate that when dNTP pools are limiting, ribonucleotides serve as a source of building blocks to maintain DNA replication. Increased levels of embedded ribonucleotides in patient cells with disturbed nucleotide pools may contribute to a pathogenic mechanism that affects mtDNA stability and impair new rounds of mtDNA replication.

## Author summary

Human mitochondria contain a small double-stranded DNA genome (mtDNA) of only 16,569 base pairs (bp) that encodes 13 essential subunits of the oxidative phosphorylation system. Depletion of mtDNA and different types of mtDNA mutations cause mitochondrial disease, and are also implicated in biological ageing. For almost half a century it has been known that mtDNA contains ribonucleotides, but their identity and precise location are not known. The source of these ribonucleotides and their relevance for mitochondrial genome stability in healthy individuals and in patients with mitochondrial defects has not been addressed. We have used a combination of next-generation sequencing, and *in vivo* and *in vitro* biochemistry to address some of these questions. Our findings demonstrate that DNA polymerase γ-dependent incorporation is the main source of ribonucleotides in mtDNA and argues against the existence of ribonucleotide excision repair pathways in human mitochondria. Our data also reveal that when dNTP pools are limiting, ribonucleotides serves as a second line of building blocks for DNA synthesis. We also demonstrate increased levels of embedded ribonucleotides in patient cells with disturbed nucleotide pools, which may constitute a new pathogenic mechanism that affects mtDNA stability and impairs later rounds of mtDNA replication.

## Introduction

Human mitochondrial DNA (mtDNA) is a double-stranded circular molecule of 16.6 kb that encodes for key components of the oxidative phosphorylation system. Previous studies have demonstrated the presence of about 10 to 30 ribonucleotides in each mtDNA molecule [1, 2]. These ribonucleotides can either be incorporated by mtDNA polymerase γ (POLγ) during DNA synthesis or, alternatively, be remnants of partially processed RNA primers used for initiation of DNA synthesis. The high levels of ribonucleotides in mtDNA are intriguing, but their precise localization or biological functions remain to be established.

Recent data suggest that incorporation of ribonucleotides during nuclear DNA replication is more frequent than previously anticipated and that the presence of these nucleotides may cause genome instability [3]. The incorporated ribonucleotides disturb the structure of DNA as they contain reactive 2´-hydroxyl groups that can attack the sugar-phosphate backbone and cause strand breaks [4]. In nuclear DNA, several processes contribute to ribonucleotide incorporation. The major replicative DNA polymerases (Pol α, Pol δ, and Pol ε) in eukaryotic cells, have all been shown to incorporate ribonucleotides during DNA synthesis [5]. Ribonucleotide incorporation into DNA may also be a consequence of inefficient removal of RNA primers used to initiate DNA synthesis. Normally, RNA primers are exchanged to DNA during RNA maturation. This process involves several nucleases, including RNase H1 and H2, Flap endonuclease 1 (FEN1), and DNA2 [6–9]. RNase H1 leaves two ribonucleotides behind and it is not clear if these two ribonucleotides are removed by further processing [10, 11]. RNase H1 has a mitochondrial isoform and deleterious mutations in the *RNASEH1* gene can disturb mtDNA replication and lead to mitochondrial disease [12].

Repair pathways exist in eukaryotes that remove incorporated ribonucleotides from nuclear DNA to prevent genomic instability. RNase H2 is required in the nucleus for ribonucleotide excision repair (RER) and mismatch repair (MMR) [13, 14]. During RER, RNase H2 nicks double stranded DNA (dsDNA) at the 5´-side of the embedded ribonucleotide. Next, Pol δ DNA synthesis leads to displacement of the incised DNA strand and the flap-cleavage by FEN1. Finally, the nicked DNA strand is ligated by DNA ligase [13]. An alternative system for ribonucleotide excision has been identified in yeast. This second system is dependent on topoisomerase 1 (TOP1), which can cleave DNA at ribonucleotides and initiate their removal [15–17]. Whether RER exists in mitochondria remains unclear, but the absence of RNase H2 argues against RER in the organelle. Mitochondria do contain a topoisomerase 1, TOP1mt, but the contribution of this enzyme to the removal of ribonucleotides from mtDNA has not been investigated [18].

The two DNA strands in the mitochondrial genome are referred to as the heavy (H) and light (L) strand, respectively, due to their different buoyant densities [18]. Each of these strands is replicated by a set of core proteins, which includes the POLγ, the mitochondrial helicase TWINKLE, the mitochondrial single stranded DNA-binding protein (mtSSB), and the mitochondrial RNA polymerase (POLRMT). According to the strand displacement model, DNA synthesis is first initiated at the origin of H-strand DNA replication (OriH) [19]. Primers required for initiation at OriH are produced by POLRMT-dependent transcription initiated at the Light Strand promoter (LSP). After initiation, replication proceeds in one direction to produce the nascent H-strand. In the process, the template H-strand is displaced and covered with mtSSB [20]. When the replication machinery has synthesized approximately two thirds of the H-strand, it reaches the origin of L-strand replication (OriL). When OriL is exposed in a single-stranded conformation, it adopts a stem-loop structure that is used by POLRMT to initiate primer synthesis from a poly-dT stretch in the loop region [21]. POLγ initiates DNA synthesis from the RNA primer and proceeds to produce the nascent L-strand using the displaced parental H-strand as template. DNA synthesis is continuous on both strands and continues until two complete daughter molecules have been formed. An alternative model for mtDNA replication has also been suggested. The Ribonucleotide Incorporation ThroughOut the Lagging Strand model (RITOLS) resembles the strand displacement model in many aspects, but suggest that poly-adenylated RNA covers the displaced H-strand, playing a role similar to that of mtSSB [22–25]. If these poly-adenylated RNA molecules covering replicating mtDNA can be used as primers for DNA synthesis remains unclear.

Pathogenic variants in both mtDNA and nuclear-encoded mitochondrial genes are a common cause of mitochondrial disease and have also been implicated as a driving force in biological aging [26, 27]. As examples, adult-onset progressive external ophthalmoplegia (PEO) and Alpers syndrome, have been linked to mitochondrial genome instability due to either multiple (variable) deletions or depletion of mtDNA [28]. The underlying causes of these mtDNA instability diseases are mutations in nuclear genes whose gene products are targeted to mitochondria. These genes can be grouped into two different classes. The first class corresponds to genes encoding proteins that are directly involved in mtDNA replication e.g. POLγA, POLγB, and TWINKLE, and the second class encodes for proteins involved in supplying mitochondria with dNTPs required for DNA synthesis, e.g. thymidine kinase 2 (TK2) [29], deoxyguanosine kinase (DGUOK) [30] and MPV17 [31, 32]. Mutations in the respective *TK2*, *DGUOK* and *MPV17* genes can lead to imbalanced dNTP pools which subsequently might lead to genome instability, however, the underlying molecular mechanisms are in most cases not fully elucidated [32–35].

In the present work, we map free 5´-ends and ribonucleotides embedded in mtDNA isolated from human control cells and fibroblasts derived from patients with disturbed dNTP pools. Our data demonstrate that mtDNA contain relative high levels of embedded ribonucleotides in both strands. In combination with a detailed biochemical analysis, our results strongly suggest that DNA polymerase γ-dependent incorporation is the main source of ribonucleotides in mtDNA and argues against the existence of a mitochondrial ribonucleotide excision repair pathway in human cells. Our study provides insights into the mechanisms of mtDNA replication during normal conditions and elucidates the consequences of limited dNTP pools associated with mitochondrial disease.

## Results

### Genome-wide 5´-end mapping and ribonucleotide mapping in mtDNA

**Fig 1:**
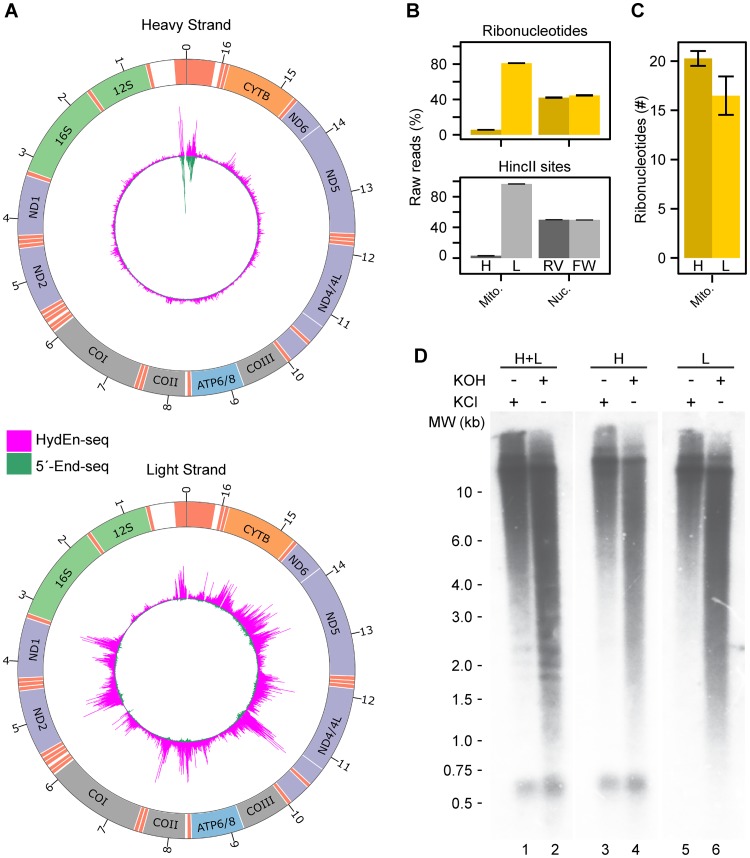
Genome-wide mapping of 5´-ends and ribonucleotides in mtDNA. (A) Circos figure of the H-strand (upper panel) and L-strand (lower panel) using 5´-End-seq and HydEn-seq. The peaks are normalized to per million reads and the maximum peak is adjusted to the maximum number of reads in the HydEn-seq library. (B) Relative amount of raw reads on the H-strand (H) or L-strand (L) in mtDNA and in the two nuclear DNA strands (reverse strand (RV) and forward strand (FW). Reads from ribonucleotide incorporations in upper panel and reads from HincII sites in lower panel. (C) Quantification of ribonucleotides in H-strand and L-strand per mtDNA molecule. (D) Southern Blot visualizing the ribonucleotide incorporation *in vivo* in mtDNA from HeLa-cells. Linearized mtDNA was subjected to salt (KCl) or alkali (KOH). Lanes 1 and 2, probes detecting both the H-strand and L-strand; lanes 3 and 4, probe detecting only the H-strand; lanes 5 and 6, probe detecting only the L-strand.

To study the distribution of ribonucleotides in mtDNA *in vivo*, we performed genome-wide mapping of HeLa cell mtDNA. In the HydEn-seq method [36], free 5´-ends are identified by combining 5´-end sequencing (5´-End-seq) with alkaline cleavage of mtDNA at embedded ribonucleotides, allowing single nucleotide resolution mapping. We identified the positions of free 5´- ends as well as incorporated ribonucleotides to the H- and L- strand (Fig 1A and S1 Fig). HydEn-seq libraries displayed a 14-fold higher coverage on the L-strand relative to the H-strand (Fig 1B, upper panel, left part). We did not observe a similar strand bias in the nuclear genome (Fig 1B, upper panel, right part). A possible reason for the observed strand bias is the distinct differences in nucleotide composition between the H- and L-strand, and to correct for such an effect we introduced eleven double strand breaks in the mtDNA by digesting genomic DNA with the restriction enzyme HincII. At these cleavage sites, we have a similar proportion of reads on the L- versus H-strand as in the uncut libraries. These reads can be used as a reference to normalize the sequencing reads (for calculation see Materials and methods and S2 Fig). Before normalization, 31-fold more reads mapped on the L-strand than on the H-strand at HincII sites, demonstrating a strong strand bias in our libraries. After normalization, we could estimate that about 20 ribonucleotides are present throughout the H-strand and about 16 ribonucleotides in the L-strand in each mtDNA molecule from HeLa cells (Fig 1C). We concluded that ribonucleotides are evenly distributed between both strands. To confirm this conclusion, we also performed Southern blotting using strand specific probes (H-strand or L-strand). In this experiment, mtDNA was linearized with BamHI and treated with KCl or KOH and separated using alkaline agarose gel electrophoresis followed by Southern blotting (Fig 1D). The control samples, treated with KCl, migrated as a band corresponding to the size of linearized full-length mtDNA (16.6 kb). The samples treated with KOH however, generated a smear of hydrolyzed products. The smears ranged from the linearized full-length product down to 7S DNA with an approximate size of 0.6 kb, suggesting that ribonucleotides are randomly distributed throughout the entire mitochondrial genome. Consistent with a previous report, the degradation patterns for the blots probed against H- and L-strand were similar, and thus supporting the even distribution of ribonucleotides between the strands of mtDNA [22].

### Location of free 5´-ends near OriH and ribonucleotides at OriL

**Fig 2:**
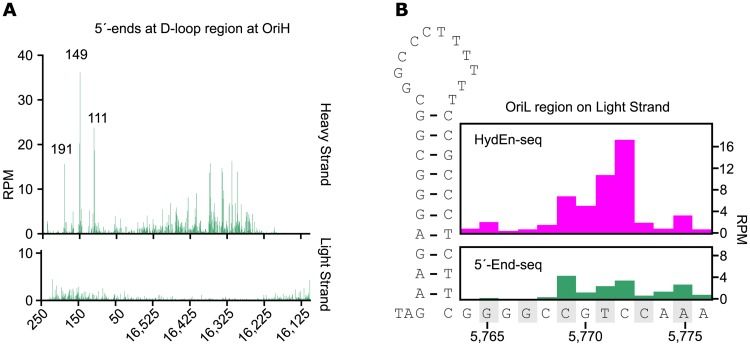
Identification of free 5´- ends at OriH and ribonucleotides at OriL. (A) Reads per million on H-strand (upper panel) and L-strand (lower panel) at OriH using 5´-End-seq, (B) Reads per million on L-strand at OriL using HydEn-seq (upper panel) and 5´-End-seq (lower panel).

Using 5´-End-seq, we found that more than 60% of the free 5´-ends in the H-strand were located near the OriH region (between positions 16,200–16,569 and 1–300) (Fig 2A, upper panel). The most abundant 5´-ends were mapped to positions 111, 149 and 191, which were consistent with previous findings [37–40]. We also observed a number of free 5´-ends in a broad, 300-nt zone, between positions 16,200–16,500 (Fig 2A, upper panel), located downstream of the OriH region. A previous report identified free 5´- ends on both the H-strand and L-strand in this region, which was used as evidence for initiation of bidirectional replication in the NCR [41]. However, our analysis revealed no free 5´-ends on the L-strand, but only on the H-strand, arguing against the idea that these ends were produced by bidirectional initiation of DNA synthesis (Fig 2A, lower panel). Furthermore, we could not observe any changes in the pattern of 5´-ends before and after alkaline treatment, arguing against the presence of attached ribonucleotides at the observed 5´-ends (S3 Fig).

On the L-strand, we identified a peak of free 5´-ends near OriL, at positions 5,768 to 5,776 (Fig 2B, lower panel). After alkaline-treatment, the 5´-ends shifted a couple of nucleotides downstream (Fig 2B, upper panel), suggesting that the identified 5´-ends contained ribonucleotides at the very end. The presence of ribonucleotides argues for RNase H1 dependent processing of the OriL RNA primer, since RNase H1 leaves the last two ribonucleotides at a junction between RNA and DNA.

### Ribonucleotide incorporation frequency by POLγ during DNA synthesis *in vitro*

**Fig 3:**
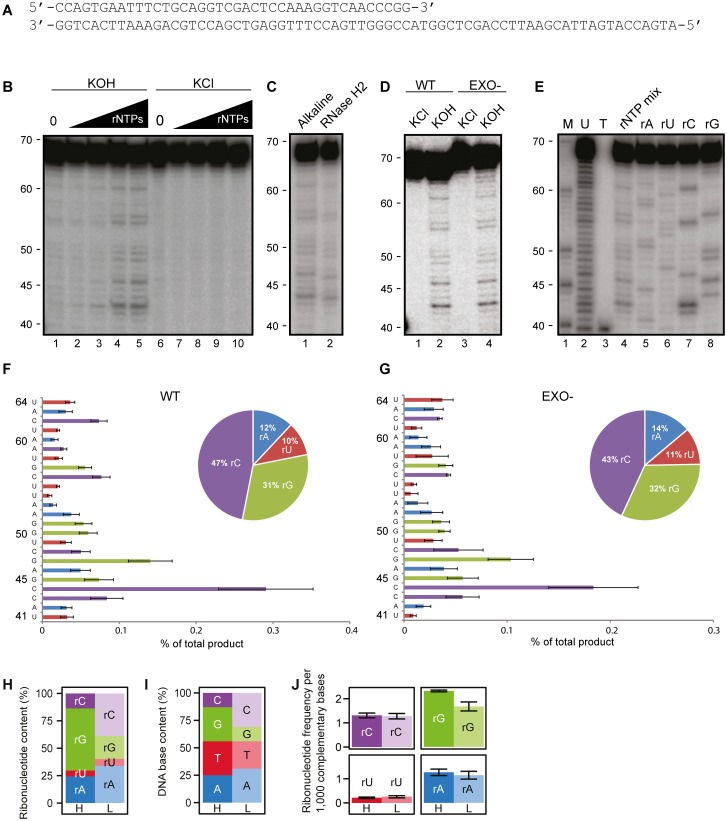
Ribonucleotide incorporation in DNA by POLγ and in mtDNA from HeLa cells. (A) Nucleotide sequence of the annealed DNA template. (B) Ribonucleotide incorporation by POLγ at increasing concentrations of all four rNTPs (0 μM, 40 μM, 100 μM, 400 μM, 1,000 μM of each) in the presence all four dNTPs (4 μM of each). Lanes 1–5, Alkali treated products; lanes 6–10, KCl treated samples (C) Cleavage products generated from alkaline and RNase H2 treatment (D) Alkaline treated or KCl treated products generated from extension assays with either POLγ or EXO-. (E) Cleavage products generated from extension assay with POLγ with the presence of indicated rNTP (4 mM) and all four dNTPs (4 μM of each). (F) Quantification of the ribonucleotide incorporation of POLγ, from the experiment in Fig 3D, as a percent of total product at every single position, inset: base composition of the incorporated ribonucleotide. (G) The same as in Fig 3F but the calculation is done with EXO- (H) Identity of incorporated ribonucleotide in H-strand (H) and L-strand (L) in HeLa cells. (I) Relative DNA base content in mtDNA of H-Strand (H) and L-strand (L). (J) Ribonucleotide incorporation frequency of an individual ribonucleotide per 1,000 complementary bases calculated on both H-strand and L-strand.

Replicative DNA polymerases can incorporate ribonucleotides during DNA synthesis and kinetic analyses of single-nucleotide incorporation events have demonstrated that mitochondrial POLγ discriminates efficiently, but not completely, against ribonucleotides [42]. To test whether POLγ could be the source of the many embedded ribonucleotides identified in mtDNA, we monitored ribonucleotide incorporation by POLγ during synthesis of longer DNA stretches *in vitro*. To this end, we generated a primed-template with a 30-nt single-stranded 5´-tail by annealing a 70-nt oligonucleotide to a complementary 40-nt oligonucleotide radioactively labeled on the 5´-end (Fig 3A). Purified recombinant POLγ was incubated with the template together with dNTPs and increasing concentrations of rNTP. Full-length products were excised, purified and subjected to alkaline hydrolysis to identify the ribonucleotide incorporation sites. As a control, we used KCl treatment, which will not hydrolyze the DNA. The levels of incorporated ribonucleotides increased with increasing rNTP concentrations (Fig 3B, lanes 1–5). The idea that alkaline sensitivity truly reflected ribonucleotide incorporation was also confirmed by processing our products using RNase H2 (Fig 3C). It is important to note that, during alkaline treatment, the extended DNA oligonucleotide will be hydrolyzed at the 3´-side, whereas RNase H2 will cleave at the 5´-side, of the embedded ribonucleotide. The product generated by RNase H2 will therefore differ by ~1 nt from the product generated by KOH treatment. We compared the ribonucleotide incorporation frequency in primer extension assays for POLγ and proofreading deficient POLγ (EXO-) when the reaction mixture contained 1 mM of each of the four rNTPs and 4 μM each of the four dNTPs (Fig 3D). These concentrations and the ratio (a 250-fold excess of rNTPs) were decided on the basis of estimates of nucleotide pools *in vivo* in mammalian mitochondria [43, 44]. In order to simplify the *in vitro* experiments, we kept the nucleotide pools balanced even though it is known that nucleotide pools *in vivo* are slightly unbalanced. We estimated product quantities from band intensities on the gels and calculated that POLγ under these conditions incorporates 1 ribonucleotide for every 2.0 ± 0.6 × 10^3^ dNTPs. We also investigated if the proofreading activity of POLγ could affect the levels of ribonucleotides incorporated during DNA synthesis. The exonuclease activity was inactivated by a D274A substitution in the second exonuclease motif in the POLγA subunit [45, 46]. Our analysis revealed that EXO- incorporates 1 ribonucleotide for every 3.0 ± 1.1 × 10^3^ dNTPs. A Student’s t-test showed no statistically significant differences in ribonucleotide incorporation between POLγ and EXO-, suggesting that the exonuclease activity of POLγ has no discernable effect on ribonucleotide incorporation *in vitro*.

Given the mtDNA size of 16.6 kb, these *in vitro* data would correspond to the incorporation of about 8 ribonucleotides in each strand during replication of mtDNA. The estimated number is in agreement with our estimates of embedded ribonucleotide levels *in vivo*, indicating that POLγ is the main driver behind ribonucleotide incorporation in human mitochondria and that an efficient mitochondrial RER pathway does not remove those ribonucleotides.

### Identification of incorporated ribonucleotides in mtDNA

Alkaline treatment of the DNA synthesized *in vitro* by POLγ generated an uneven band pattern on the gel (Fig 3B, 3C and 3D), suggesting that the frequency of ribonucleotides incorporation is sequence dependent. To precisely map the observed sites, we performed *in vitro* DNA synthesis with the same template, but with only one type of ribonucleotide added at the time (Fig 3E). In this manner, we could verify the incorporation pattern of individual ribonucleotides. The DNA template used in our experiments contained roughly equivalent numbers of the four bases (30% A, 23% C, 27% T, 20% G). From reaction mixtures containing POLγ or EXO-, we quantified the band intensities and calculated the mean frequency and standard error from three independent experiments (Fig 3F and 3G). Both POLγ and EXO- generated similar incorporation pattern, again demonstrating that the proofreading activity does not affect ribonucleotide incorporation during DNA synthesis. Of the four ribonucleotides, rCTP was the most frequently incorporated into the newly synthesized strand, followed by rGTP, whereas rATP and rUTP were less frequently incorporated (Fig 3F, inset and Fig 3G, inset). Our data were thus in agreement with a previous single nucleotide incorporation study, which demonstrated that POLγ is less efficient in discriminating against rCTP and rGTP compared to rATP and rUTP [42].

To follow up these observations, we determined the relative frequencies of each individual ribonucleotide in mtDNA from HeLa cells *in vivo*. Again, we focused our analysis on positions 300–16,200 of mtDNA and excluded the OriL region from 5,747–5,847. The H- and L- strands had different profiles of embedded ribonucleotides (Fig 3H). Whereas rGTP was the most frequent ribonucleotide in the H-strand it was only the third most frequent in the L-strand. Similarly, rCTP was the most frequent ribonucleotide in the L-strand, but it was the third most frequent in the H-strand, whereas rUTP was the least frequent ribonucleotide in both strands. Mitochondrial DNA has a higher proportion of guanine (G) in the H-strand and cytosine (C) in the L-strand (Fig 3I). We investigated if the strand bias seen in rGTP and rCTP frequencies could be explained by the strand bias in DNA base composition in mtDNA. Using HydEn-seq data from HeLa cells and the DNA base composition in mtDNA, the raw reads for each individual rNTP were normalized to obtain incorporation frequencies per 1,000 complementary nucleotides in mtDNA (Fig 3J, see Materials and methods for details). In this normalized data, we do not observe strand bias between the two strands in terms of which rNTP is incorporated. There is a preferential incorporation of rGTP on both strands, intermediate levels of rCTP and rATP, and low levels of incorporated rUTP. The frequency of incorporation for individual ribonucleotides was therefore in nice agreement with the *in vitro* biochemical profile of POLγ-dependent ribonucleotide incorporation (Fig 3 and [42]). The only exception was the frequency of rATP incorporation, which was somewhat higher than expected based on *in vitro* biochemical experiments. This deviation may indicate that the relative concentration of rATP present in mitochondria is higher than what was used in our *in vitro* assays. Taken together, these results are consistent with POLγ incorporation being the main contributor to the incorporation of ribonucleotides in mtDNA.

### Changes in nucleotide pools influence ribonucleotide incorporation *in vivo*

If POLγ incorporates ribonucleotides during DNA synthesis, one would expect that altered or unbalanced mitochondrial dNTP and rNTP pools could affect ribonucleotide incorporation frequencies and profiles, as observed *in vitro* (Fig 3B). A number of reported pathogenic mutations causing mitochondrial disease have been linked to unbalanced mitochondrial nucleotide pools and, as a consequence, mtDNA instability. To test whether disturbed nucleotide pools leads to changes in ribonucleotide incorporation rates in mtDNA *in vivo*, we performed HydEn-seq using patient-derived cell lines harboring recessively-inherited pathogenic variants in the *TK2*, *DGUOK* or *MPV17* genes. For *TK2*, we used three different patient cell lines and for both *DGUOK* and MPV17 we used two different cell lines, each harboring distinct recessively-inherited pathogenic gene variants (details provided in Materials and methods).

**Fig 4:**
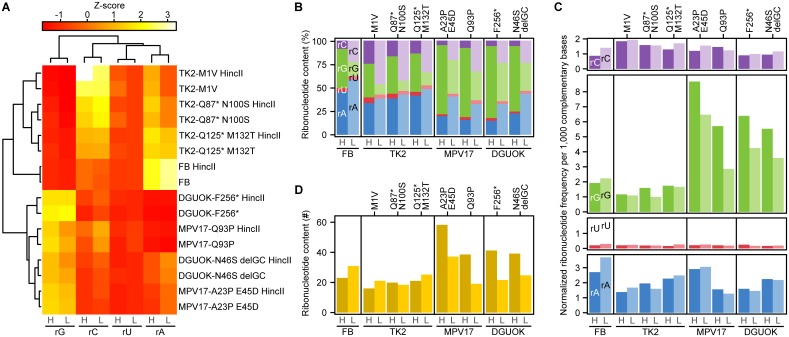
Ribonucleotide incorporation in mtDNA from fibroblasts from patients with genetic defects in *TK2*, *DGUOK* or *MPV17*. (A) Hierarchical clustering of HydEn-seq libraries (undigested or digested with HincII). (B) Ribonucleotide content for H-strand (H) and L-strand (L) for patient-derived mutant cell lines. (C) Ribonucleotide incorporation frequency per 1,000 bases of its complementary nucleotides calculated on H-strand and L-strand for fibroblasts and patient-derived mutant lines. (D) Quantification of ribonucleotides in H-strand and L-strand per mtDNA molecule in fibroblasts and patient-derived mutant lines.

We first investigated if the mtDNA ribonucleotide profiles of the mutant fibroblasts differed from that of controls by performing hierarchical clustering on z-scores calculated from the ribonucleotide incorporation percentages (Fig 4A). For each of the cell lines, sequencing reads from libraries made with undigested or HincII-digested DNA cluster together, showing that the treatment does not change the detected overall ribonucleotide distribution. Two main cell line clusters can be observed, one with the control Fibroblast (FB) and cells with *TK2* mutations and the other with pathogenic *DGUOK* and *MPV17* variants. The FB and *TK2* mutant clusters are characterized by relatively high incorporation of rATP and rCTP levels whereas the *DGUOK* and *MPV17* mutant clusters have relatively high incorporation of rGTP. The ribonucleotide profile of TK2-Q125* M132T is intermediate to FB and the other *TK2* mutant lines, indicating that this compound heterozygous variant may have a milder phenotype (Fig 4A).

The patient fibroblasts displayed changes in ribonucleotide incorporation that were consistent with the underlying pathological mutations (Fig 4B). Fibroblasts with mutations in genes encoding DGUOK or MPV17 have decreased mitochondrial dGTP pool size, and in our experiments, the relative levels of embedded rGTPs were increased relative to the other ribonucleotides. Fibroblasts with mutations in the gene encoding TK2 have a decreased dCTP pool, and correspondingly the relative incorporation of rCTP is increased in our experiments. The incorporation rates of rATP, rCTP and rGTP appeared to be different on the H- and L-strand (Fig 4B). Here we again normalized the data to obtain incorporation frequencies per 1,000 complementary DNA bases (Fig 4C, see Materials and methods for details). After this data normalization we could no longer observe any strand bias, indicating that the difference seen in incorporation percentages (Fig 4B) can be explained by the DNA base composition and not due to a different replication mode in each strand.

The rCTP incorporation frequency was somewhat elevated in TK2 compared to FB whereas the rGTP incorporation frequencies were 2 to 3-fold higher in DGUOK and MPV17 compared to FB. All three mutant lines appear to have a somewhat lower rATP incorporation frequency. All four lines showed very low rUTP incorporation frequencies. We conclude that imbalanced mitochondrial dNTP pools change the pattern of rNTP incorporation in a manner consistent with the biochemical properties of POLγ (Fig 4C).

Next, we calculated the number of ribonucleotides in each strand in each of the cell lines and found that the fibroblast cells have a slight increased ratio (23 ribonucleotides in H-strand and 31 in L-strand) compared to HeLa cells (Fig 4D). Fibroblasts from controls and patients with defects in TK2 contain slightly more ribonucleotides in the L-strand, while the patients with defects in DGUOK and MPV17 have more ribonucleotides in the H-strand. The increased number of ribonucleotides in DGUOK and MPV17 in the H-strand may be a direct consequence of changes in rGTP:dGTP ratio in the combination with the high G content in the H-strand. Similarly cell lines with defects in TK2 have changed rCTP:dCTP ratio and increased incorporation frequency of rCTP on the L-strand, which has a high C content.

## Discussion

In the current report, we use a genome-wide approach to map the distribution and identity of free 5´-ends and embedded ribonucleotides *in vivo* with single-nucleotide resolution. We find that mtDNA isolated from HeLa cells on average contains 36 embedded ribonucleotides (Fig 1C) with similar levels in mtDNA from fibroblasts (54 ribonucleotides per genome, Fig 4D). The number of incorporated ribonucleotides is almost evenly distributed between the H- and L-strand in mtDNA from both HeLa cells and fibroblasts (Figs 1C, 1D and 4D). The fact that equal levels of ribonucleotides are embedded in both strands also argues against the idea that these nucleotides represent remnants from priming events during DNA replication. Priming at other sites than OriH and OriL would take place during L-strand synthesis (i.e. lagging strand) and if these priming events left embedded ribonucleotides behind, we would expect to see higher levels in the L-strand (Fig 1C).

The relative levels of the individual ribonucleotides embedded *in vivo*, correlates well with *in vitro* observations using recombinant POLγ to investigate the incorporation of ribonucleotides during synthesis of longer DNA stretches (Fig 3F). Ribonucleotide rCTP is most frequently incorporated, followed by rGTP, whereas rATP and rUTP are less efficiently incorporated. These observations are also consistent with a previous report from the Copeland laboratory, which used single-nucleotide extension assays to demonstrate that the frequency of ribonucleotide incorporation is dependent on the base [42]. POLγ is much more efficient in discriminating rUTP from dTTP (77,000-fold) than rGTP from dGTP (1,100-fold). The ribonucleotide incorporation by POLγ *in vitro* depends on the rNTP/dNTP ratio, and under the conditions used here 1 rNTP is incorporated for every 2,000 dNTPs. This rate is similar to what was reported for the three replicative DNA polymerases in *S*. *cerevisiae*, where pol ε incorporates 1 ribonucleotide for every 1,250 dNTPs, pol δ 1 rNTP for every 5,000 dNTPs, and pol α 1 rNTP for every 625 dNTPs [5]. The rNTP:dNTP ratio also affects the relative levels of ribonucleotide incorporation *in vivo* (Fig 4A and 4B). Mutations in *DGUOK* or *MPV17* lead to depletion of dGTP and, as demonstrated here, result in increased rGTP incorporation during mtDNA synthesis (Fig 4C, green panel). Mutations in *TK2* cause a decrease in dCTP, which in turn cause increased incorporation of rCTP (Fig 4C, purple panel).

Our data also reveal a difference in primer maturation between OriL and OriH. At least two ribonucleotides remain associated with the free 5´-end at OriL (characteristic of RNase H1 processing). The free 5´-ends observed at OriH do not contain associated ribonucleotides. These findings support the idea that RNase H1 cannot be the only nuclease required for primer processing at OriH, but that this process involves more complex mechanisms and additional enzymes, including MGME1 [9, 40, 47–49]. We also map additional free 5´-ends in a broad, 300-nt zone, between positions 16,200–16,500 (Fig 2A, upper panel). These ends have previously been seen as an indication of bidirectional replication, but since they are only present on the H-strand, we speculate that the observed ends instead represent intermediates in 7S DNA breakdown. Being the third strand of the D-loop, 7S DNA strand is synthesized at much higher levels than other regions of the mtDNA genome, but how this strand is degraded after displacement have not been characterized [50]. Further experiments are clearly required to define the nature of the observed 5´-ends in the 16,200–16,500 region.

POLγ does not seem to proofread ribonucleotides as the inactivation of the exonuclease activity fail to cause higher levels of rNTP incorporation *in vitro* (Fig 3D). Likewise, neither yeast pol ε nor human pol δ can proofread ribonucleotides [51, 52]. A certain level of embedded ribonucleotides seems to be well tolerated by the mtDNA replication and transcription machineries. It has been shown that POLγ efficiently performs single-nucleotide reverse transcription reactions but that longer stretches of embedded ribonucleotides in template DNA cause POLγ stalling [42]. Our genome-wide mapping of ribonucleotides also does not support the existence of longer stretches of ribonucleotides in the mtDNA, which can cause a problem for mtDNA maintenance. However, we cannot rule out that mutations in e.g. *TK2*, *DGUOK* or *MPV17* may cause imbalanced ribonucleotide pools in specific tissues, which can lead to the formation of longer stretches of embedded ribonucleotides, which in turn may cause problems during mtDNA replication and transcription. Further efforts to perform tissue specific HydEn-seq analysis may address this interesting possibility.

Finally, embedded ribonucleotides may play functional roles in DNA maintenance. In support of this notion, ribonucleotides in nuclear DNA help to identify the nascent DNA strand during MMR. When RNase H2 removes ribonucleotides during RER, nicks are formed in the nascent strand, and the MMR system can thereby distinguish which strand to correct [14]. Possible mitochondrial processes regulated by embedded ribonucleotides remain to be identified, but we however conclude that when dNTP pools are limiting, ribonucleotides may serve as a second line of building blocks for mtDNA synthesis. Without ribonucleotide incorporation, cells may suffer more acute mtDNA instabilities due to replication stalling.

## Materials and methods

### Patients

Primary skin fibroblast cultures were obtained from healthy age-matched controls and patients with confirmed pathogenic variants in one of three genes implicated in disorders of mtDNA maintenance, *TK2*, *DGUOK* and *MPV17*, leading to mtDNA depletion myopathy (patients with *TK2* variants) or hepatocerebral mtDNA depletion (patients with *DGUOK* and *MPV17* variants). The following patient cells were studied: TK2-M1V (homozygous p.(Met1Val) *TK2* variant (Patient 25 in [53]); TK2-Q87* N100S (compound heterozygous p.(Gln87*); p.(Asn100Ser) *TK2* variant (Patient II-4 in [54]); TK2-Q125* M132T (compound heterozygous p.(Gln125*); p.(Met132Thr) *TK2* variants (Garone *et al*, manuscript under review)); DGUOK-N46S delGC (compound heterozygous p.(Asn46Ser); c.13_14delGC *DGUOK* variants); DGUOK-F256* (homozygous p.(Phe256*) *DGUOK* variant (Patient 10 in [55]); MPV17-A23P E45D (compound heterozygous p.(Ala23Pro); p.(Glu45Aspfs*8) *MPV17* variants (Patient 2 in [56]); MPV17-Q93P (homozygous p.(Gln93Pro) *MPV17* variant (Patient 11 in [56]). Ethical approval was granted by the Newcastle and North Tyneside Local Research Ethics Committees (REC 2002/205), the study was performed under the ethical guidelines issued by each of our institutions and complied with the Declaration of Helsinki.

### DNA isolation from HeLa and patient fibroblast cells

Cells were grown in 70 ml of DMEM GlutaMAX medium (Gibco), 10% fetal bovine serum (Gibco) in 250 ml Spinner flasks (Bellco Glass Inc.). A total of 5x10^6^ cells were collected by centrifugation for 5 min at 200 × g and washed once with PBS. Pellets were resuspended in 2 ml of lysis buffer (75 mM NaCl, 50 mM EDTA, 1% SDS, 20 mM HEPES pH 8.0, 200 μg/ml Proteinase K) and incubated at 42°C for 30 min. One volume of phenol-chloroform was added to the samples, then mixed and centrifuged at 15,000 × g for 5 min (4°C). The water phase was then transferred to a new tube for precipitation with 100 mM NaCl and 1 V of isopropanol. Samples were incubated at -20°C at least for 1 h. After precipitation, the samples were centrifuged (15,000 × g, 20 min, 4°C) and washed with 70% EtOH. Pellets were dissolved in 100 μl of TE buffer. DNA concentrations for library preparations, was measured with a Qubit fluorometric instrument (ThermoFisher Scientific). Patient and control fibroblast cells were grown in DMEM GlutaMAX medium, supplemented with 10% FBS, PEST and 50 μg/ml uridine. Between 4 and 11 × 10^6^ cells were collected and total DNA was isolated using the Gentra Puregene Cell Kit (Qiagen) according to the manufacturer´s protocol.

### Mapping of free 5´-ends and ribonucleotides *in vivo*

Free 5´-ends in mtDNA of HeLa and primary fibroblast cells were mapped by 5´-End-seq by treating 1 μg DNA with 0.3 M KCl for 2 h at 55°C. RNA residues in mtDNA of HeLa and primary fibroblast cells were mapped by HydEn-seq by hydrolyzing 1 μg DNA with 0.3 M KOH for 2 h at 55°C. To calculate the number of ribonucleotides per mtDNA molecule, we treated 1 μg DNA with 10 U of HincII and the digests were purified with HighPrep PCR beads (MagBio), before the KCl or KOH treatment. After ethanol precipitation, the DNA fragments were treated for 3 min at 85°C, phosphorylated with 10 U of 3′-phosphatase-minus T4 polynucleotide kinase (New England BioLabs) for 30 min at 37°C, heat inactivated for 20 min at 65°C and purified with HighPrep PCR beads (MagBio). Phosphorylated products were treated for 3 min at 85°C, ligated to oligo ARC140 overnight at room temperature with 10 U of T4 RNA ligase, 25% PEG 8000 and 1 mM CoCl_3_(NH_3_)_6_, and purified with HighPrep PCR beads (MagBio). Ligated products were treated for 3 min at 85°C. The ARC76–ARC77 adaptor was annealed to the second strand for 5 min at room temperature. The second strand was synthesized with 4 U of T7 DNA polymerase (New England BioLabs) and purified with HighPrep PCR beads (MagBio). Libraries were purified, quantified with a Qubit fluorometric instrument (ThermoFisher Scientific) and 50-base paired-end sequenced on an Illumina NextSeq500 instrument, to identify the location of the free 5´-ends.

### Sequence trimming, filtering, and alignment

All reads were trimmed for quality and adaptor sequence with cutadapt 1.2.1 (-m 15 -q 10–match-read-wildcards). Pairs with one or both reads shorter than 15 nt were discarded. Mate 1 of the remaining pairs was aligned to an index containing the sequence of all oligos used in the preparation of these libraries with bowtie 0.12.8 (-m1 -v2), and all pairs with successful alignments were discarded. Pairs passing this filter were subsequently aligned to the hg38 *H*. *sapiens* reference genome (-m1 -v2 -X10000–best). Single-end alignments were then performed with mate 1 of all unaligned pairs (-m1 -v2). Using the–m1 setting causes Bowtie to discard all reads which align to multiple places in the genome, including nuclear mitochondrial DNA segments (NUMTs). To calculate the base identity of ribonucleotides in mtDNA, the count of 5′-ends of all paired-end and single-end alignments were determined for all samples and shifted one base upstream to the location of the free 5′-end or hydrolyzed ribonucleotide. For visualizing reads aligning to the entire mitochondrial genome in Fig 1, the pipeline described above was used with the difference that the–m1 flag was not used. The counts were normalized to reads per million (RPM) and visualized using Circos software.

### Estimation of ribonucleotides per mitochondrial molecule

To estimate the number of ribonucleotides per mitochondrial molecule the mtDNA cut with HincII was used. For each of the H- and L strands, the reads at each position were first normalized to reads per million. Subsequently, the mean number of reads at the eleven HincII sites, including reads five positions upstream and downstream thereof, was calculated. The reads from these double-stranded breaks give a relative quantification of all of the mitochondrial molecules. Dividing the total reads in the region between 300 and 16,200 and excluding the OriL region from 5,747–5,847 for each strand, not including reads at the eleven HincII sites, with the mean number of reads gives the number of ribonucleotides per single strand break, i.e. the number of ribonucleotides per mitochondrial molecule.

### Calculation of ribonucleotide incorporation frequency

To calculate ribonucleotide incorporation frequency, we made use of HydEn–seq libraries. For each of the H- and L- strands, the number of reads in the region between 300 and 16,200 and excluding the OriL region from 5,747–5,847 for each of the four ribonucleotides was normalized to the mean number of reads in HincII sites. This gives the number of each individual ribonucleotide per mitochondrial molecule. This number was converted to incorporation frequency by dividing by the total number of the complementary nucleotides in mtDNA (in the region between 300 and 16,200 and excluding the OriL region from 5,747–5,847) and scaled by a factor of 1,000.

### Heatmaps, hierarchal clustering

For each strand and library the relative incorporation percentage of each ribonucleotide was calculated. In samples treated with HincII the reads mapping to those recognition sites were excluded prior to calculating percentages. The percentage data was then re-scaled on a per strand and ribonucleotide basis to a z-score with a mean of 0 and a standard deviation of 1. Hierarchical clustering was performed on the re-scaled data using the “heatmap” function from the “stats” package in R.

### Southern blot

HeLa cells were trypsinized followed by wash with PBS. Cells were resuspended in lysis buffer (10 mM Tris-HCl pH 8.0, 0.1 M NaCl, 25mM EDTA pH 8.0 and 0.5% SDS) and incubated at 55°C for 2 hours followed by phenol extraction and ethanol precipitation to isolate DNA. Samples were resuspended in TE buffer (10 mM Tris-HCl pH 8.0, 1 mM EDTA) overnight. Total DNA (24 μg) was digested with BamH1-HF and thereafter supplied with 0.3 M NaCl and 300 ng RNaseA to remove single-stranded RNA. The DNA was precipitated and resuspended in TE buffer and aliquoted into two tubes. The samples were treated with either 0.3 M KCl or 0.3 M KOH, for 2 h at 55°C in a hybridization oven and then aliquoted into three and run on a 0.8% alkaline agarose gel. The samples were transferred to a nylon Hybond-N+ membrane (GE Healthcare) and UV-crosslinked. Strand specific probes (L-strand or H-strand) against the human mtDNA molecule were hybridized to the DNA as indicated in the figure legends. Probe sequences are available from the authors upon request.

### Recombinant proteins

Human recombinant POLγA and POLγB (WT and EXO- versions), were expressed and purified as described previously [46].

### Incorporation of ribonucleotides on a short, linear DNA template

A 70-nt oligonucleotide (5´-ATG ACC ATG ATT ACG AAT TCC AGC TCG GTA CCG GGT TGA CCT TTG GAG TCG ACC TGC AGA AAT TCA CTG G-3´) was annealed to a 40-nt DNA oligonucleotide (5´-CCA GTG AAT TTC TGC AGG TCG ACT CCA AAG GTC AAC CCG G-3´) labeled in the 5´-end with [γ-^32^P] ATP to produce a primed-template that can be used as a substrate for DNA polymerization [57].

The reaction mixture (20 μl) contained 600 fmol of the DNA template, 25 mM Tris-HCl, pH 7.8, 1 mM DTT, 10 mM MgCl2, 100 μg/ml BSA, 4 μM dNTP, 600 fmol of WT or EXO- POLγA, 1200 fmol POLγB and each NTP to final concentrations of; 0 μM, 40 μM, 100 μM, 400 μM, 1 mM, or 4mM rATP, rUTP, rCTP and rGTP respectively. The reaction was incubated at 37°C for 30 min and stopped by the addition of 20 μl formamid loading buffer (95% formamid, 25 mM EDTA, 10 mg/ml bromophenol blue, 10 mg/ml xylene cyanol). The samples were loaded on a 7 M urea, 8% polyacrylamide sequencing gel in 1 × TBE buffer. Full-length products were excised from the gel and precipitated with ethanol precipitation and resuspended in TE buffer. The products were counted in a scintillation counter and equal amount of counts from the samples were mixed with either KOH or KCl to a final concentration of 0.3 M. The samples were incubated at 55°C for 2 hours or treated with RNase H2 at 37°C for 1 hour. The reactions were stopped by addition of formamide loading buffer and analyzed by electrophoresis on a 7 M urea, 8% polyacrylamide sequencing gel in 1 × TBE buffer and signals were visualized by autoradiography.

Quantification was performed in MultiGauge and the results are the average from three independent experiments.

## Supporting information

S1 Fig Spearmans coefficient was calculated for H-strand (HS) and L-strand (LS) in HydEn-seq and 5´-End-seq libraries for mtDNA isolated from HeLa cells. (PDF)

S2 Fig Summarized signal at HincII sites in mtDNA in L-strand (upper left panel), mtDNA in H-strand (lower left panel), nuclear DNA in top strand (upper right panel), nuclear DNA in bottom strand (lower right panel). (PDF)

S3 Fig 5´-End-seq and HydEn-seq results at OriH. (PDF)
