# Supplementary figures and images for: Nucleotide pools dictate the identity and frequency of ribonucleotide incorporation in mitochondrial DNA
Source: PLoS Genet. 2017 Feb 16;13(2):e1006628. doi: 10.1371/journal.pgen.1006628 (PMC5336301; doi:10.1371/journal.pgen.1006628)

Supplementary Figure 1

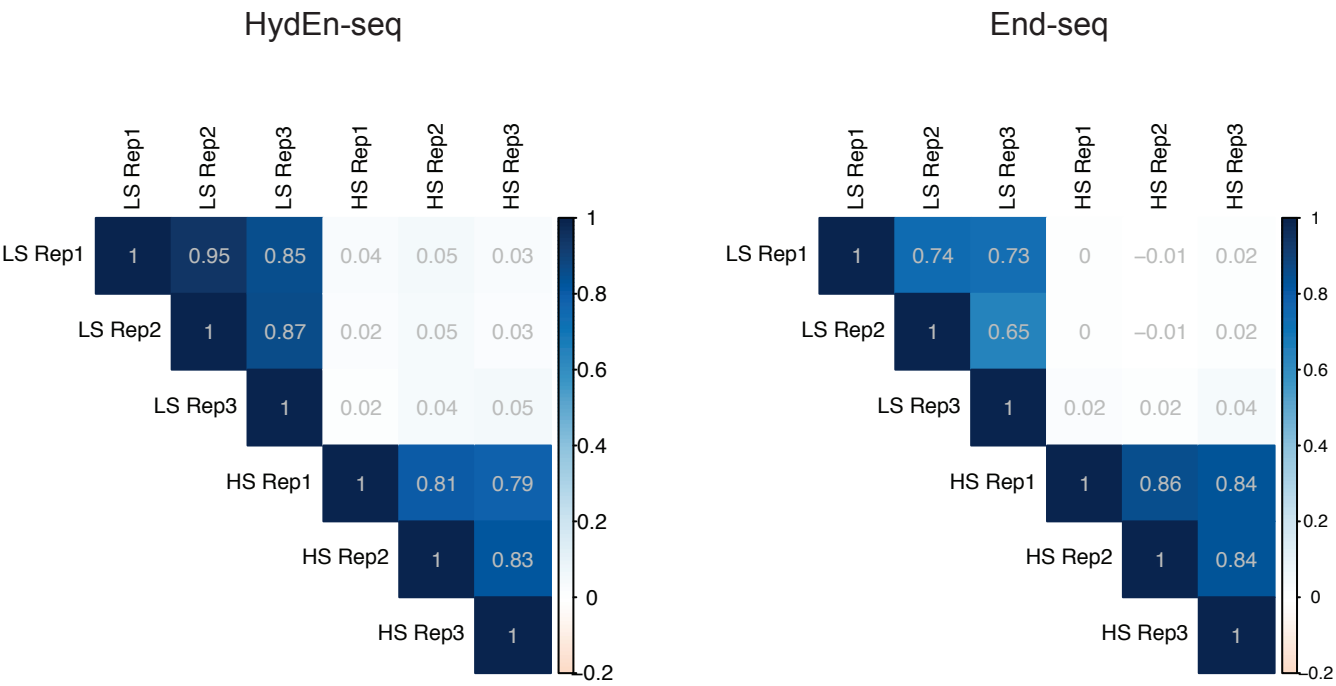

Supplement: S1 Fig — (PDF) [file pgen.1006628.s001.pdf]

Supplementary Figure 2

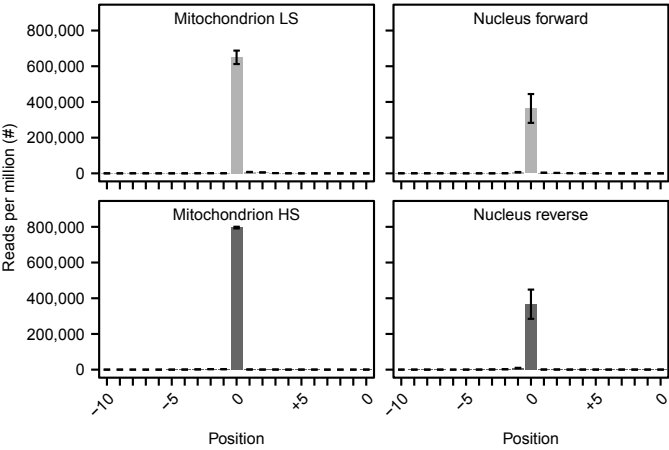

Supplement: S2 Fig — (PDF) [file pgen.1006628.s002.pdf]

Supplemetary Figure 3

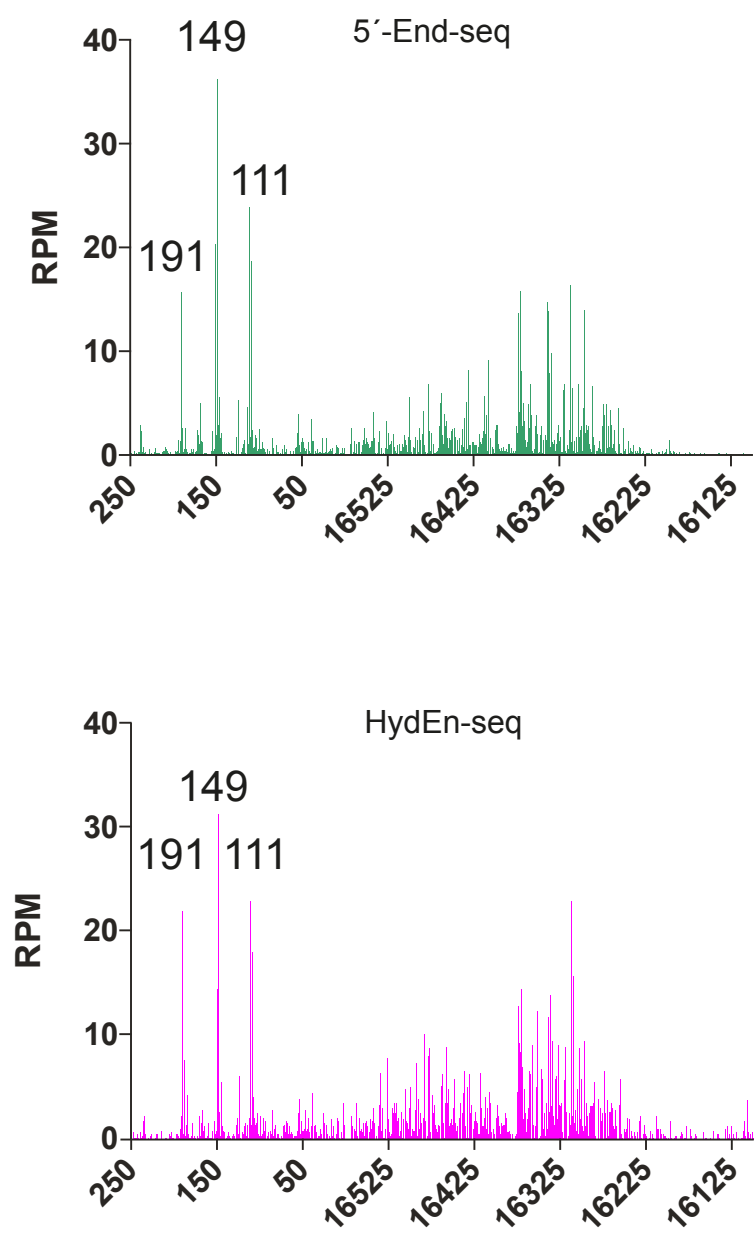

Supplement: S3 Fig — (PDF) [file pgen.1006628.s003.pdf]
